# The Animal Rights Debate: Abolition or Regulation?

**DOI:** 10.3390/ani1010200

**Published:** 2011-03-15

**Authors:** Ben Mepham

**Affiliations:** Centre for Applied Bioethics, School of Biosciences, University of Nottingham, Sutton Bonington Campus, LE12 5RD, UK; E-Mail: ben.mepham@nottingham.ac.uk

The simple, but quite unusual plan, promised much: two leading academics (Gary Francione and Robert Garner) prepared to make their respective cases with conviction and candour, followed by a lively debate between them, during which no stone need be left unturned. In prospect, then, was an opportunity for nuances to be clarified, misunderstandings dispelled and some common ground established. But while it proved to be an enthralling engagement, the apparent inability of one author to concede even an inch of ground to the other's case meant that the divide between the viewpoints was, regrettably, no narrower at the end than at the beginning.

Francione, a law professor at Rutgers University in the USA, is a leading philosopher of animal rights theory. He has pioneered the abolitionist theory of animal rights, claiming that animal welfare regulation is both theoretically and practically unsound, and serves merely to prolong the status of animals as property by making the public feel comfortable about using ‘happy’ animals. Accordingly, abolishing animal use, e.g., by adopting strict veganism, is the only acceptable course of action and the only basis on which people ought to be encouraged to relate to animals.

Garner, a politics professor at Leicester University, UK, is a political theorist who specialises in the field of animal protection. One of his major concerns (as summarised on his website) is ‘the difficulties of protecting the interests of animals from within the liberal tradition: focusing on the costs, for liberal societies, of adopting animal rights; the impact of liberal neutrality as regards the good society; and the protection of human autonomy’. While acknowledging the importance of campaigning for veganism, and indeed practising it personally, he considers it can realistically only form one part of an overall program to encourage more ethical treatment of animals.

The contents of the book fall into three, roughly equal, parts, viz. Francione's essay, ‘The abolition of animal exploitation’, Garner's essay, ‘A defence of a broad animal protectionism’, and the discussion between the two. In essence, both the essays and the discussion epitomise the marked differences that prevail between a lawyer's categorical, legalistic, approach and the much more pragmatic, politically-informed, approach of a political scientist. In the following, reference to specific points is made by quoting in parentheses the pages on which they are made.

Francione's position is based on several strong assertions. For example, he claims that: ‘ethical veganism is a recognition of the moral personhood of animals’, for which the only cognitive characteristic required is that they be sentient (p. 15), a condition which is itself inconsistent with the property status of animals, and with any animal use, however ‘humane’ (p. 4). While conceding that there are ‘relevant differences between the minds of humans and non-humans’, these are not logically relevant to whether they are used in experiments or killed for other purposes (p. 19), because ‘to say that a sentient being is not harmed by death denies that the sentient being has the very interest that sentience serves to perpetuate’ (pp. 15,16). These rights arguments are supported by several consequentialist claims, e.g., that animal welfare regulations do not work, and that there is ‘absolutely no evidence whatsoever’ that animal welfare reform will reduce animal use in future (p. 26); that ‘most human disease is due to consumption of animal products’ (p. 29), which are also a disaster for the environment (p. 28); and that welfarism has not decreased animal use (p. 47)—in fact, animal advocacy groups have aided the meat industry (p. 46).

Unsurprisingly, the categorical nature of such assertions encounters some difficult issues. The thorny question of how to cope with the needs of billions of non-humans on a shrinking planet is answered by arguing that we should stop bringing animals into existence (p. 79), an objective which is facilitated by the belief that ‘sterilisation is consistent with abolitionist programs’ (p. 80). For ‘however well we treat non-human companions, they are completely dependent on humans for every aspect of their existence’ (p. 79). And in response to the alleged inconsistency that vegans appear unconcerned by the harms accidentally inflicted on wildlife in cultivating crops or in building roads, Francione argues that although it is impossible to avoid such secondary effects, they are ‘different morally from intentionally killing an individual animal’ (p. 72).

In conclusion, Francione argues that bringing about a paradigm shift in our relationship with animals would be best achieved by a program of ‘clear, unequivocal, non-violent education targeted at abolishing all animal use’ (p. 85). For, if instead of the dominant welfarist approach, an abolitionist agenda had been pursued in the past, ‘there likely would be many hundreds of thousands more vegans than there are today’ (p. 65).

Garner's approach is quite different in style and in the assumptions made about how improvements in the condition of non-humans might best be achieved. For him: ‘it is important to distinguish between what is prescribed by ethics and what is achievable politically or strategically, because any viable moral discourse must take into account more than rationalistic ethical principles’ (p. 105). Like Francione, he stresses that sentience is a crucial factor in ascribing ethical standing to animals, but this does not equate with the notion that animals have ‘personhood’ (p. 113) For example, loss of life causes harms to humans that, inter alia, entail loss of beliefs, desires, goals, projects *etc.*—experiences of a qualitatively different nature from those experienced by animals (p. 116).

In answer to Francione's claim that animal welfare measures introduced have been counter-productive because people feel more comfortable about eating meat from ‘happier animals’, Garner argues that the improvements are in themselves desirable, and that claims about the likely effectiveness of vegan campaigns are highly speculative (p. 122): indeed, it is possible that matters might be worse if all efforts had been directed to an abolitionist program. The political reality is that no country in the world has prohibited use of animals in medical research or as a source of food (p. 125). Garner agrees that much of what is currently permitted under the aegis of animal welfare ought to be banned (p. 119), but suggests that ‘no measure that does not at the same time promote benefit to humans is likely to gain a hearing’ (p. 126).

Fundamentally, he argues that abolitionist aims are unrealistic because the underlying principle—that animals have a moral status close, if not identical, to humans—is not accepted by the vast majority of society (p. 143). The political reality for Garner is that ‘the battle of ideas in a liberal democratic society is conducted in a pluralistic universe where the rationale is not for one set of ideas to prevail but for a plurality of ideas to coexist’ (p. 148), conditions which, e.g., have been the guiding force behind post-war legislation on homosexuality in some western societies. In this context, he cites the late John Rawls, who stressed the importance, in seeking sound political decisions, of ‘reflective equilibrium’, *i.e.*, the need to check rational principles with moral intuitions. The upshot is that, for him, the advantage of concentrating on eliminating unnecessary suffering is that it maximises public support, and is progressive in that what is deemed ‘unnecessary’ has expanded over recent decades, reflecting greater public awareness, changes in cultural norms and technological developments (p. 142).

Although the discussion is presented as a verbatim dialogue, it would be interesting to know precisely how it was conducted. For example, were the discussants present in the same room or conversing by telephone? Did the discussion occupy more than one session? Since the published version is word perfect, one suspects it was edited—and if so it presumably lost some spontaneity, and hence authenticity. Even so, the discussion is a revealing part of the book, although perhaps not entirely in ways the protagonists might have anticipated.

Without attempting a blow-by-blow summary of the exchange, I doubt that I am the only reader to be left with an impression of a discussion characterised by Francione's somewhat strident approach, to which Garner responded with remarkable amiability. And while Garner was prepared to acknowledge several areas of agreement, Francione's only reciprocal response was the single word ‘agreed’, in the book's final sentence, to the proposition that currently animals endure unacceptable levels of suffering. Not even the revelation that Garner was a dietary vegan, in reply to Francione's ‘personal’ question, elicited any recorded response (p. 257). A discussion where one participant seems unprepared to concede any merit in the opposing perspective, a condition which is perhaps intrinsic to an absolutist, abolitionist stance, hardly provides fertile ground for intellectual enquiry. Yet it might have been anticipated that the fact that both participants reputedly sought to achieve desired changes incrementally would have provided a sound basis for fruitful discussion.

Readers of this review will doubtless detect a bias in views expressed here, but there are some matters (perhaps like the appropriateness of nudism, or belief in astrological predictions!) which it is virtually impossible for a thoughtful person to approach with a completely open mind. Acknowledging that caveat, Francione seems to me to be guilty of making several assertions that are either speculative in the extreme or based on a very limited consideration, or understanding, of objective evidence.

One example that must suffice to make a point of more general validity is his claim that ‘Animal agriculture is a disaster for the environment because it involves a very inefficient use of natural resources …’ (p. 28). While the statement is accurate in the case of many intensive animal production systems, it is certainly not universally true. Thus, the claim ignores those, admittedly rarely-practised, forms of animal husbandry (but how can vegans dismiss claims merely because they are made by other minorities?), which operate within a permaculture livestock economy, and that achieve efficient food productivity in socially and ecologically integrated systems, in which ‘the by-product of one component is easily available to provide the feedstock of another’. The quotation is from Simon Fairlie's book [1], which might be read with advantage by vegans, not least because Fairlie's aim was to provide ‘both a critique of veganism and a tribute to vegans and vegetarians from Shelley onwards…’.

Fairlie's point assumes enormous significance in terms of the environmental crisis that humanity faces in consequence of the combined effects of climate change and diminishing fossil fuel reserves; developments that imply the urgent need to adopt sustainable practices entailing ‘energy descent’. In achieving the latter, energy will necessarily be largely derived from biomass, renewable resources such as wind, water and solar, and increased use of muscle power; and farm animals could play an effective role in different elements of this process.

The Farncione/Garner book certainly provides clear expositions of contrasting attitudes, but the territory explored often seems rather abstract, omitting consideration of several critical issues. So in conclusion it is perhaps appropriate to identify some of these. For example, the ethical implications of the following surely merit attention.

(i) The resources allocated to concern for animals as opposed to those employed to address human survival, suffering or wellbeing—which may entail substantial opportunity costs for vegans. For example, the energy and time expended in ensuring dietary integrity, and promoting the vegan cause, is necessarily denied to efforts to ease the suffering of humans, of whom about one billion suffer from malnutrition in less-economically developed countries, while many, world-wide, are victims of abuse and exploitation.
(ii) The rights and interests of humans at the level of personal health and wellbeing, in societies whose economies, cultures and, in some cases survival, depend on a symbiotic relationship with animals. The discussion in the book seems dominated by attitudes and conditions in western ‘developed’ countries.
(iii) The fate of prey, and more generally of adverse effects on ecological sustainability in the wild (including animal and plant species in managed wildlife reserves), of permitting predators, and wild species in general, to satisfy their biological needs. If sentience is the sole relevant characteristic in granting animals rights, what could be the ethical basis of showing partiality to domesticated rather than wild animals?
(iv) The adverse effects on wild animals (at individual, group and species levels) of practices employed in arable farming, on which most vegan diets are necessarily dependent. For example, pest control, not only of insects but also of indisputably sentient mammals like rabbits and mice, is crucial for efficient arable farming. Is it really valid to argue that since the adverse effects on wild animals are ‘accidental’ they are beyond vegans' ethical concern?
(v) The basis of the distinction assigned to the meaning of ‘property’ in the cases of farmed animals (especially when kept in organic, and particularly permacultural, systems) and ‘rescued’ companion animals. Indeed, it is pertinent that there is a real sense in which human children are, when young, their parents' ‘property’—without this being considered remotely problematical from an ethical perspective.

The rigour with which vegans attempt to lead lives according to the strict observance of ethical principles is often regarded as exemplary, and vegan philosophy certainly represents a significant challenge to long-established norms of behaviour. But if the aim of ethics is to choose the right, or best, course of action in specific circumstances ‘all things considered’, it is arguable that adherence to such an absolutist agenda is simplistic and open to serious self-contradictions. Or, as Farlie puts it, with characteristic panache: ‘to conclude that veganism is the ‘only ethical response’ is to take a big leap into a very muddy pond’ (p. 2).
